# Spread of a New Parasitic B Chromosome Variant Is Facilitated by High Gene Flow

**DOI:** 10.1371/journal.pone.0083712

**Published:** 2013-12-26

**Authors:** María Inmaculada Manrique-Poyato, María Dolores López-León, Josefa Cabrero, Francisco Perfectti, Juan Pedro M. Camacho

**Affiliations:** 1 Departamento de Genética, Facultad de Ciencias, Universidad de Granada, Granada, Spain; 2 Departamento de Células Troncales, Centro Andaluz de Biología Molecular y Medicina Regenerativa (CABIMER), Sevilla, Spain; University of Massachusetts, United States of America

## Abstract

The B_24_ chromosome variant emerged several decades ago in a Spanish population of the grasshopper *Eyprepocnemis plorans* and is currently reaching adjacent populations. Here we report, for the first time, how a parasitic B chromosome (a strictly vertically transmitted parasite) expands its geographical range aided by high gene flow in the host species. For six years we analyzed B frequency in several populations to the east and west of the original population and found extensive spatial variation, but only a slight temporal trend. The highest B_24_ frequency was found in its original population (Torrox) and it decreased closer to both the eastern and the western populations. The analysis of Inter Simple Sequence Repeat (ISSR) markers showed the existence of a low but significant degree of population subdivision, as well as significant isolation by distance (IBD). Pairwise *N_e_m* estimates suggested the existence of high gene flow between the four populations located in the Torrox area, with higher values towards the east. No significant barriers to gene flow were found among these four populations, and we conclude that high gene flow is facilitating B_24_ diffusion both eastward and westward, with minor role for B_24_ drive due to the arrival of drive suppressor genes which are also frequent in the donor population.

## Introduction

B (supernumerary or accessory) chromosomes are dispensable elements found within the chromosome complement of about 15% of eukaryotes [1]. They frequently behave as genome parasites, harming host fitness and prospering in natural populations because they show drive (i.e. transmission rates higher than the Mendelian one). B chromosomes typically show irregular meiosis since their number is not restricted to two, and they do not always form a bivalent segregating one B to each meiotic pole, which is the usual situation for standard (A) chromosomes.

B chromosomes are dynamic genome elements and their frequency in populations varies as a result of factors such as the intensity of drive, effects on the host genome and even historical factors [1]. Theoretical studies have shown that B chromosome invasion is a very rapid process, lasting only some tens of generations, whereas the subsequent stages (neutralization by the host genome and near-neutral loss) are longer [2]. Rapid invasions have been reported in the grasshopper *Eyprepocnemis plorans* [3], [4], the fish *Prochilodus lineatus* [5] and the wasp *Trypoxylon albitarse* [6], [7]. In *E. plorans*, B chromosomes have been found in almost all the populations hitherto analyzed within the subspecies *E. plorans plorans* around the Mediterranean Sea and the Caucasus [8], the only exception being several populations at the head of the Segura river [9].

One of the most remarkable features of the B chromosome polymorphism in *E. plorans* is that new B chromosome variants emerge regularly and replacements of one variant for another is common [1]. In the Torrox population (East Málaga), a replacement of B_2_ by a larger new variant (B_24_) could be witnessed directly and was based on significant drive for B_24_ [3]. Whereas B_24_ frequency has remained very high in later samples of this population, the average transmission ratio of this B chromosome variant declined to 0.51 in only six years [10]. The finding of B_24_ in the Algarrobo population, located about 9 km west of Torrox, suggested that this new B variant is currently expanding from its original nucleus [11]. Towards the east, however, the only population sample previously analyzed at Nerja [12] showed no trace of the B_24_ chromosome, but recent samples shown in this paper have shown that it has also arrived to this population. In order to follow this spreading process, we documented spatial and temporal patterns of B chromosome variants from the Torrox and surrounding populations over a period of six years. This may be important, not only for disentangling the evolutionary history of these genomic elements but also to understand their evolutionary role as genome parasites.

To obtain indirect estimates of gene flow between these populations, we analyzed Inter Simple Sequence Repeat markers (ISSR) in five populations. As ISSR markers evolve quickly, they provide a large number of polymorphic markers [13] that are very useful for the genotypic identification of individuals, even those closely related, and for studies on population structure in non-model species where genomic information is rather scarce, as in the case of the grasshopper *E. plorans*. In a previous study, ISSR markers revealed the existence of high gene flow among *E. plorans* populations [14]. In this paper, we analyze the evolution of the temporal and spatial frequency of a new B chromosome variant (B_24_), and perform laboratory crosses to tract the inheritance of these elements, in the populations surrounding Torrox, i.e. its putative center of origin. The analysis of ISSR markers provided an estimate of gene flow and population structure that help us to understand the ongoing spread of new B-chromosome variants. Whereas B chromosome drive explains the rapid B invasion in a given population, reaching other populations by B chromosomes depends, as in other strictly vertically transmitted parasites, on the existence of gene flow in the host species. Here we show that gene flow in *E. plorans* is high enough to explain the spread of the newly arisen B_24_ variant.

## Results

**Figure 1:**
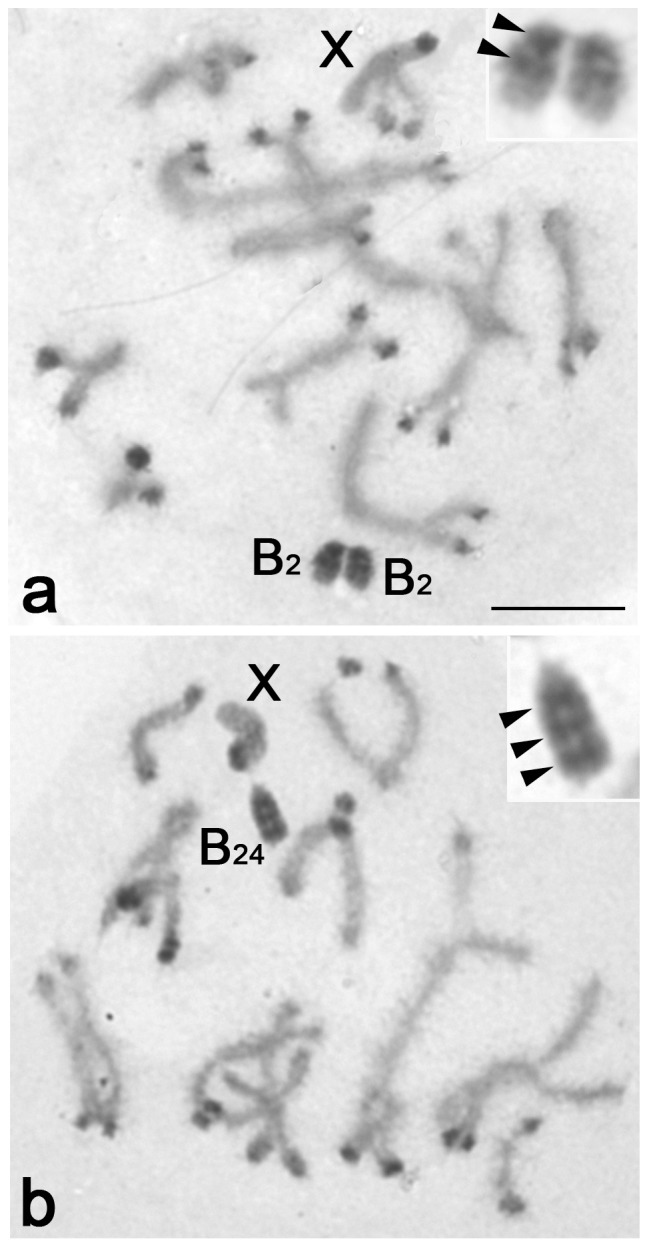
C-banded primary spermatocytes at pachytene-diplotene showing the B_2_ (a) and B_24_ (b) chromosome variants. Arrowheads point the presence of two (inset in a) and three (inset in b) dark C-bands in B_2_ and B_24_, respectively.

Cytological analysis showed the presence of two types of B chromosomes, corresponding with the B_2_ and B_24_ variants previously described in this species [3], [15]. The C-banding technique showed the presence of two dark C-bands in B_2_ and three in B_24_ (Fig. 1).

### Temporal and Spatial Analysis of B Chromosome Variation

Table 1 shows the frequency of B chromosomes found in the six populations analyzed over several years, expressed as the proportion of B-carrying individuals (prevalence) and the mean number of B chromosomes per individual (mean). The observed variation appeared to be stochastic in five populations, with only Nerja-0, i.e. the population closest to Torrox, showing B_2_ frequency decrease approaching significance (Table 2). In fact, a comparison between 2001 and 2006 in this population showed that prevalence for B_2_ decreased significantly (P = 0.037, SE = 0.001) during this six-year period in which B_24_ prevalence passed from 0.087 to 0.205 (see Table 1).

**Table 1:** Frequency of B chromosomes expressed as prevalence (P) and mean number of B chromosomes per individual (M) in six natural populations of the grasshopper *Eyprepocnemis plorans* collected in Málaga province (Spain) over several years.

			Prevalence						Mean				Mean				Mean
Population	Year	N	B_2_	B_24_	All Bs	0B_2_	1B_2_	2B_2_^+^	B_2_	0B_24_	1B_24_	2B_24_^+^	B_24_	0B	1B	2B^+^	All Bs
Algarrobo	2001	21	0.143	0.19	0.333	18	3	0	0.143	17	4	0	0.19	14	7	0	0.333
	2002	108	0.278	0.222	0.407	78	29	1	0.287	84	22	2	0.241	64	31	13	0.528
	2003	77	0.195	0.312	0.429	62	15	0	0.195	53	22	2	0.338	44	25	8	0.532
	2004	76	0.211	0.303	0.461	60	16	0	0.211	53	18	5	0.368	41	27	8	0.579
	2005	61	0.262	0.18	0.361	45	15	1	0.279	50	6	5	0.262	39	15	7	0.541
	2006	33	0.303	0.182	0.394	23	10	0	0.303	27	6	0	0.182	20	10	3	0.485
Torrox	2004	61	0.082	0.705	0.738	56	3	2	0.115	18	22	21	1.115	16	21	24	1.23
	2006	44	0.045	0.818	0.864	42	2	0	0.045	8	20	16	1.341	6	22	16	1.386
Nerja-0	2001	23	0.609	0.087	0.652	9	8	6	0.913	21	2	0	0.087	8	8	7	1
	2004	63	0.381	0.095	0.476	39	21	3	0.429	57	5	1	0.111	33	26	4	0.54
	2006	44	0.318	0.205	0.523	30	10	4	0.432	35	9	0	0.205	21	19	4	0.636
Nerja-1	2001	12	0.583	0.083	0.583	5	6	1	0.667	11	1	0	0.083	5	5	2	0.75
	2004	54	0.463	0.056	0.481	29	22	3	0.519	51	3	0	0.056	28	21	5	0.574
	2006	47	0.362	0.149	0.511	30	13	4	0.447	40	6	1	0.17	23	19	5	0.617
Nerja-2	2001	28	0.464	0	0.464	15	10	3	0.571	28	0	0	0	15	10	3	0.571
	2004	46	0.522	0.043	0.543	22	20	4	0.674	44	2	0	0.043	21	20	5	0.717
	2006	35	0.629	0	0.629	13	19	3	0.714	35	0	0	0	13	19	3	0.714
Maro	2001	27	0.333	0	0.333	18	9	0	0.333	27	0	0	0	18	9	0	0.333
	2004	19	0.158	0.053	0.211	16	3	0	0.158	18	1	0	0.053	15	4	0	0.211
	2006	16	0.313	0	0.313	11	4	1	0.438	16	0	0	0	11	4	1	0.438

N =  Total number of individuals analyzed. Individuals with two or more B chromosomes were grouped into the same category (2B^+^), but the means were calculated with the ungrouped data.

**Table 2:** Analysis of temporal variations in the prevalence of B chromosomes.

		Prevalence						
		B_2_		B_24_		All Bs		
	Distance	P	SE	P	SE	P	SE	
Algarrobo	8.45	0.562	0.018	0.355	0.02	0.836	0.008	
Torrox	0	0.696	0.003	0.251	0.007	0.144	0.004	
Nerja-0	4.84	0.058	0.004	0.235	0.007	0.356	0.01	
Nerja-1	6.46	0.3	0.007	0.29	0.007	0.854	0.003	
Nerja-2	8.89	0.411	0.009	-	-	0.438	0.011	
Maro	10.37	0.432	0.008	-	-	0.675	0.004	

Distance =  km from Torrox. P and SE are the probability value for the RXC contingency analysis, and the standard error of P, respectively.

**Figure 2:**
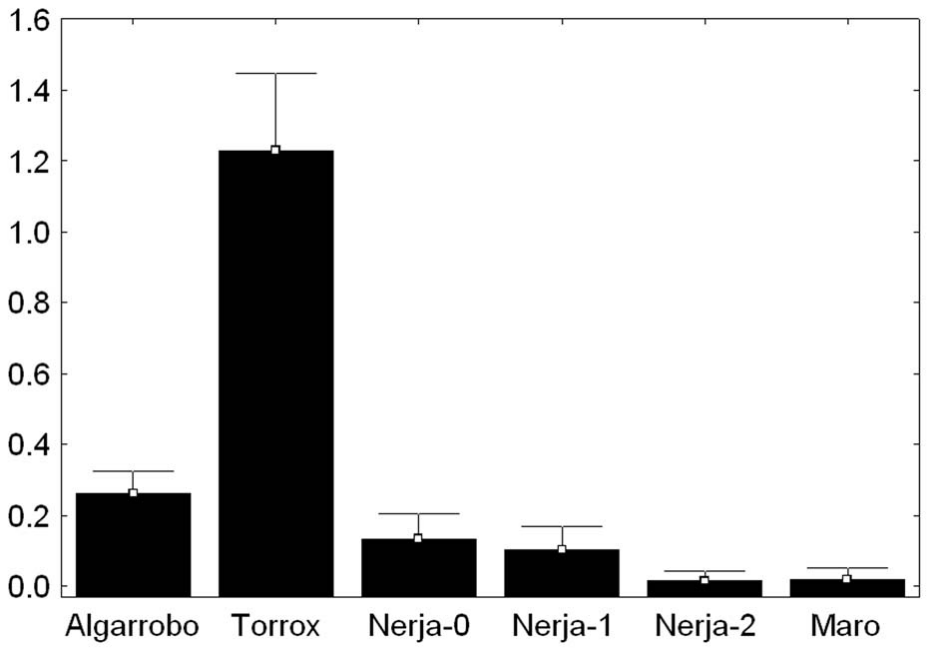
Mean frequency of B_24_ chromosomes in five localities near Torrox in Málaga province (Spain), showing how this B chromosome variant that emerged in Torrox is diffusing both eastward and westward. Populations are displayed from the west (Algarrobo) to the east (Maro). Frequencies were averaged per population including all year samples analyzed. Note that individuals can carry more than one B chromosome and thus mean B frequency can be higher than 1, as actually occurs in Torrox.

Significant spatial variation was found among populations for B_2_ and B_24_ prevalence and mean (contingency chi-square tests: P<0.000001, SE<0.000001). B_24_, the invading B variant, showed a contagious distribution pattern (Fig. 2), with the highest frequency at Torrox (its center of origin), low frequencies at Algarrobo and Nerja-0 (the populations closest to Torrox, to the west and east, respectively), a slightly lower frequency at Nerja-1, located a mere 1.6 Km from Nerja-0, and only sporadic appearances in more distant populations (Nerja-2 and Maro) (Table 1). Spearman non-parametric correlation tests showed a marginally significant negative correlation between the geographical distance to the center of origin and the mean frequency of the B_24_ chromosomes found in 2004 (*r_s_* = −0.77, *t* = −2.43, *P* = 0.07), but this association was significant for the 2006 sample (*r_s_* = −0.93, *t* = −4.97, *P* = 0.0077). This result is consistent with the existence of isolation by distance.

### Transmission of B Chromosome Variants

To ascertain whether geographical patterns of B chromosome frequency variation could be explained by differences in B chromosome transmission between populations, we performed 16 controlled crosses in the laboratory. In total, we performed 12 progeny analyses for the B_2_ chromosome and 9 for the B_24_ variant, which included the cytological analysis of 32 parents and 332 embryo offspring. As a whole, most crosses showed transmission ratios for B chromosomes not significantly different from the Mendelian one (K_B_ = 0.5). The only exceptions were two crosses from Algarrobo (f13 × m20 and f26 × m21) that showed a significant deficit of B_2_ chromosomes transmitted via the male parent, one cross from Algarrobo (f4 × m19) showing significant B_24_ drive via the female parent, and one cross from Nerja-0 (f2 × m4) showing significant B_24_ elimination (Table 3). As a whole, the 12 crosses for B_2_ and the nine for B_24_ showed average transmission ratios (0.49 and 0.47, respectively) being very close to the Mendelian one (0.5). However, B_24_ transmission rate showed higher variation between individuals (SD = 0.15) than B_2_ (SD = 0.11).

**Table 3:** Transmission of B_2_ and B_24_ in controlled crosses.

					Embryo offspring									
		Parents			B_2_					B_24_				
Population/year	Cross	Male	Female	Total embryos	Mean	B_p_	P_B_	k_B_	Z	Mean	B_p_	P_B_	k_B_	Z
Algarrobo/2005	f4 × m19	0B	1B_24_	29						0.690	1	f	0.690	2.04
	f8 × m34	0B	1B_2_+2B_24_	11	0.455	1	f	0.455	-0.30	1.000	2	f	0.500	0.00
	f12 × m13	0B	1B_24_	15						0.600	1	f	0.600	0.77
	f18 × m19	0B	1B_2_+2B_24_	8	0.625	1	f	0.625	0.71	0.875	2	f	0.438	-0.35
	f16 × m14	1B_2_	0B	23	0.478	1	m	0.478	-0.21					
	f10 × m18	1B_2_+1B_24_	0B	21	0.667	1	m	0.667	1.53	0.381	1	m	0.381	-1.09
	f13 × m20	1B_2_	0B	32	0.313	1	m	0.313	-2.12					
	f26 × m21	1B_24_	0B	24						0.250	1	m	0.250	-2.45
	f27 × m23	1B_24_	0B	35						0.571	1	m	0.571	0.85
Nerja-0/2005	f1 × m5	0B	1B_2_	11	0.545	1	f	0.545	0.30					
	f2 × m4	2B_2_	1B_2_+1B_24_	21	1.667	3	f,m	0.556	0.51	0.238	1	f	0.238	-2.40
	f3 × m3	1B_2_	0B	20	0.500	1	m	0.500	0.00					
Nerja-1/2004	f3 × m9	0B	1B_2_+1B_24_	31	0.355	1	f	0.355	-1.62	0.516	1	f	0.516	0.18
	f5 × m7	0B	1B_2_	19	0.368	1	f	0.368	-1.15					
	f11 × m19	0B	2B_2_	18	1.000	2	f	0.5	0.00					
	f18 × m13	0B	1B_2_	14	0.500	1	f	0.5	0.00					

f =  female, m =  male, Bp =  number of Bs in the parents, PB =  parent carrying Bs, k_B_ =  mean transmission ratio for the B chromosome, Z =  Z-test indicating B-drive if higher than 1.96, or B-drag if lower than −1.96. Significant Z-tests are indicated in bold-type letter.

### Population Genetic Analysis with ISSR Markers

In the sample as a whole (Table S1), 97% of the ISSR loci analyzed were polymorphic whereas, per population, this figure was 77.6% on average (standard deviation: SD = 3.05) (78% in Algarrobo, 81% in Torrox, 80% in Nerja-0, 75% in Nerja-2 and 74% in Salobreña).

A significant population structure was evident, as shown by θ^(I)^ = 0.291±0.026 (mean ± SD) (analogous to Wright's *F_ST_*), θ^(II)^ = 0.058±0.007 (analogous to Nei's G_ST_), and G_ST_-B = 0.049±0.004 (which is a Bayesian estimate of G_ST_). The expected heterozygosity per population (hs) was 0.235±0.005 in Algarrobo, Torrox and Nerja-2, 0.225±0.005 in Nerja-0 and 0.236±0.005 in Salobreña. The average expected heterozygosity (Hs) for the five populations was 0.233±0.003, and that for the five populations calculated from mean allele frequencies was Ht = 0.245±0.003. Only one private allele was found, i.e. 39-1000 in the Algarrobo population.

**Figure 3:**
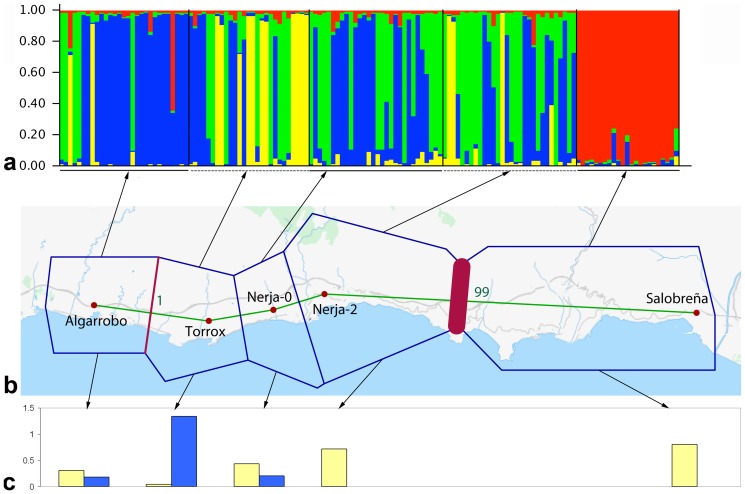
Genetic and geographical structure of the *E. plorans* populations analyzed. a) Ancestry of each individual from the four genetic groups (K = 4), using all 94 fragments of different size observed for the six ISSR markers analyzed, yielded by the Structure software. Each vertical bar represents one of the 139 individuals analyzed. Note that all individuals from Salobreña were assigned to group 4 (red), most individuals from Algarrobo were assigned to group 1 (blue) whereas individuals from Torrox and Nerja showed a mixture of groups 1 (blue), 2 (green) and 3 (yellow). Bar length is proportional to the ancestry values inferred in each group for each individual. b) Geographical location of the five populations used for the ISSR analysis, Delaunay triangulation and the significant barrier (red) between Nerja-2 and Salobreña populations, detected by the Barrier software. c) Mean number of B chromosomes per individual for the B_2_ (yellow) and B_24_ (blue) variants, found in 2006, i.e. the same year in which the ISSR markers were analyzed.

The number of ancestral population groups (*K*) deduced from the Evanno *et al*. [16], method was 4 (Fig. S1). With *K* = 4, the Structure software showed that Salobreña and, to a lesser extent, Algarrobo were the most differentiated populations, in accordance with their geographical distance. The three remaining populations (Torrox, Nerja-0 and Nerja-2) showed more mixed patterns, in keeping with their greater proximity (Fig. 3a, Table S2).

The effective number of migrants per generation (*N_e_m*) between population pairs (Table 4), which is an indirect measure of gene flow, was 4 on average, suggesting that population divergence by genetic drift is hampered by a high rate of gene flow. A Mantel test comparing the *N_e_m* and geographical distance matrices showed significant IBD (R = −0.894, P = 0.017), thus indicating that the effective number of migrants decreases with geographical distance. The IBD analysis was also performed by a regression analysis of log-transformed *N_e_m* values on log-transformed kilometers [17] (Table S3). A significant negative correlation was observed between these two variables (r = −0.889, P = 0.0005), also suggesting the existence of significant IBD. The existence of IBD was confirmed by the Mantel test performed with the genetic distances obtained by Dice's method (R = 0.731, P = 0.025), thus indicating that genetic dissimilarity between populations increases with geographical distance.

**Table 4:** Pairwise *N_e_m* (above diagonal) and *F_ST_* (below diagonal) values observed in the five populations analyzed for the ISSR markers.

	Algarrobo	Torrox	Nerja-0	Nerja-2	Salobreña
Algarrobo		5.5	7.6	4.7	2.8
Torrox	0.0432		8.8	10.0	2.6
Nerja-0	0.0320	0.0276		12.1	2.6
Nerja-2	0.0501	0.0243	0.0203		2.0
Salobreña	0.0814	0.0884	0.0888	0.1136	

The Barrier software consistently detected a single significant barrier between Salobreña and the other populations, but no significant barriers to gene flow were found between the populations surrounding Torrox (Fig. 3b). The geographical distribution of B chromosome frequency for both B_2_ and B_24_, found in the same year as ISSRs were analyzed (2006), showed that B_24_ is most frequent in Torrox (its center of origin), and that this chromosome has reached the adjacent populations of Algarrobo (to the west) and Nerja (to the east), but it was not found in more distant populations such as Nerja-2 and Salobreña (Fig. 3c).

## Discussion

The presence of two B chromosome variants in the *E. plorans* populations analyzed, one of which (B_24_) emerged recently in the Torrox population as a derivative from the other variant (B_2_) [3], [18], provides a unique opportunity for analyzing the spread of B_24_ and thus witnessing evolution in action. We previously witnessed the increase of B_24_ frequency at the expense of B_2_ one, due to significant drive for the former [3]. As our present data shows, this new B variant had already reached the adjacent Algarrobo and Nerja populations by 2001 (see Table 1). Whereas no previous sample had been analyzed in Algarrobo, a sample from the Nerja population analyzed by Henriques-Gil et al. [12] showed the presence of the B_2_ chromosome in 22 out of 43 males analyzed, with no traces of B_24_. We can therefore assume that B_24_ arrived in Nerja after 1984.

Our present results show little temporal variation for B chromosome frequency. This suggests that changes in B frequency are mostly stochastic and do not show a clear tendency to increases in B_24_ frequency, or else that it is too slow to capture in the six year time span conducted here. The spatial analysis, however, revealed very significant differences between populations, with B_24_ being most frequent in Torrox, i.e. its putative center of origin, and progressively less frequent to the east and west, thus suggesting that this new B variant is expanding its geographical range by diffusion toward nearby populations. The absence of significant temporal changes in B_24_ frequency is expected from the absence of net drive in the nine crosses performed for this variant, despite the extensive variation observed between crosses. It is also consistent with the idea that the diffusion of genes through a metapopulation is a very slow process [19]. This slowness is explained, among other reasons, by the random movement of individuals (and genes) in space, and also because the immigrant genes do represent a very low proportion of total genes in the receptor population. A more efficient means of spreading genes is the colonization of new places, or the recolonization of others in which con-specific individuals became extinct because, in these cases, the immigrant genes represent up to 100 per cent. Shaw [20] claimed that the latter kind of spreading explained the rapid movement of a B chromosome cline observed in a ten-year period in the grasshopper *Myrmeleotettix maculatus*. In *E. plorans*, the B_24_ variant appears to be rather young (only a few decades) and its spread might have been somewhat influenced by the recent agricultural and tourist development of the Torrox area.

The analysis of ISSR markers has provided valuable information on genetic variation, population structure, gene flow and isolation by distance (IBD) in *E. plorans*.

The low but significant θ^(II)^ and G_ST_-B values (both about 0.05) indicated some population subdivision. This was reinforced by the Structure analysis, which showed the existence of four genetic groups, although only the one including most individuals from Salobreña was well defined, the remaining showing much admixture due to high gene flow (Table 4). *N_e_m* values were high in the four populations in Málaga province (4.7–10), suggesting a greater intensity of gene flow than of genetic drift [21]. When *N_e_m*>4, a metapopulation is usually considered to be behaving like a panmictic population [22]. These high values could actually be somewhat biased since they were calculated from *F_ST_*, which is dependent on sample size and allele frequency [23]. In fact, *F_ST_* values below 0.02 (and several of our values come close) lead to *N_e_m* estimates that may be influenced by sampling noise [24]. As pointed out by Waples [23], the difficulty in measuring low *F_ST_* values makes it difficult to get good estimates of *N_e_m* in species with a high dispersal capability. However, as pointed out by Palumbi [24] when the genetic data show a marked IBD, as observed in *E. plorans*, the *F_ST_* estimates can be considered more robust. The significant IBD shown by the Mantel test thus allows concluding, with greater confidence, that genetic differentiation in these *E. plorans* populations is the result of biological characteristics. Furthermore, *N_e_m* values were calculated on the basis of the islands model [25], with a similar migration rate (m) between every two islands. Even though this model appears to be unreal, especially in our case, with the unidimensional stepping-stone distribution of the populations analyzed (see Fig. 3), Slatkin & Barton [26] showed that *N_e_m* estimates under the island model are of the correct order of magnitude but depend on the geographical distance between populations. In sum, the ISSR marker analysis supports the existence of enough population connectivity to facilitate the spread of these chromosome variants to nearby populations.

B chromosome invasions have occasionally been witnessed, and they always appeared to be very rapid [3]–[7]. Remarkably, drive suppression also appears to be rapid in *E. plorans*, since the transmission ratio of the B_24_ chromosome in the Torrox population decreased from 0.7 to 0.5 in only six years [10]. Elsewhere, the B chromosomes in the fish *Prochilodus lineatus* [5] and the wasp *Trypoxylon albitarse* [6], [7] showed signs of rapid stabilization, suggesting some kind of neutralization by the host genome [1]. The existence of drive suppressor genes was first suggested by Shaw [27] and was later demonstrated in several organisms [28]–[35]. The rapid suppression of drive for B_24_ in Torrox [10] was presumably due to an increase in the frequency of drive suppressor genes in this population, which were advantageous due to the significant decrease in egg fertility in females carrying B_24_ [3]. As pointed out by Shaw [27], suppressor genes might serve other purposes and may have already existed in the population at a low frequency.

Why was B_24_ invasion so fast in Torrox while its diffusion to nearby populations is so slow?

B_24_ frequency in Torrox reached a maximum in 1994 (1.53 Bs per individual, on average) [3], and it declined in subsequent years in parallel to drive suppression occurred by 1998 [10]. Therefore, migration of Torrox individuals to adjacent populations (Nerja and Algarrobo) exported both B_24_ chromosomes and the gene variants suppressing its drive, which had already reached high frequency in Torrox. This explains why the average transmission rate for B_24_ found in Algarrobo in 2004 (0.6) (see Table 6 in Manrique-Poyato *et al*. [11] was lower than that found in Torrox in 1992 (0.7) [3]. Remarkably, transmission rate for B_24_ showed extensive variation in Algarrobo females (0.38–0.92), suggesting the existence of polymorphism for drive suppression, a fact that is also apparent in the present crosses (see Table 3). The average transmission rates of B24 in Algarrobo and Nerja indicate that drive currently plays a minor role in B_24_ spread to other populations.

The spread of a new gene or chromosome variant could occur through diffusion or else through the colonization of empty sites. As explained above, one clear difference between the two processes is that the diffusion of genes between populations is a slow process whereas the recolonization of empty habitats is faster [19]. In *E. plorans*, the populations from Málaga province, analyzed here, inhabit places next to somewhat similar cultivations. Therefore, we believe that the B_24_ spread towards B_2_-carrying populations is taking place through diffusion, rather than through recolonization. Furthermore, the high efficiency of B chromosome drive suppression in *E. plorans*, which rapidly neutralizes B chromosomes [10], [36], [37] could significantly contribute to the slowing down of the spread of B_24_ chromosomes, which is thus mostly dependent on high gene flow.

## Materials and Methods

During the years 2001 to 2006, a total of 895 adult male and female *Eyprepocnemis plorans* were collected from six natural populations on the coast of Málaga (Spain), namely Torrox and five other populations, one to the west (Algarrobo) and four (Nerja-0, Nerja-1, Nerja-2 and Maro) to the east (Table 1). Note that out of Nerja populations, Nerja-0 was the closest to Torrox and Nerja-2 the farthest from it. In addition, 332 embryos were analyzed from 16 controlled crosses performed with specimens from the populations located adjacent to Torrox, i.e. Algarrobo (specimens collected in 2005), Nerja-0 (2005) and Nerja-1 (2004), to study B chromosome transmission. No specific permits were required for these field studies. The locations sampled were not privately owned or protected in any way, and this field study did not involve endangered or protected species.

The males were anaesthetized in ethyl acetate vapours before dissection to extract the testes, which were fixed in 3∶1 ethanol-acetic acid and stored at 4°C. The females were injected abdominally with 0.1 ml of 0.05% colchicine in insect saline solution for 6 h before anesthesia and dissection to extract ovaries, which were fixed in 3∶1 ethanol-acetic acid and stored at 4°C. Embryos were dissected out of the eggs, after a ten-day incubation period at 27°C, and fixed for cytological analysis following Camacho *et al.* [38]. B chromosome variant identification was performed on C-banded preparations performed as in Camacho *et al.* [38]. Microscopic digital photographs were obtained on a DP70 Olympus camera coupled to an Olympus BX41 microscope. The photographs were optimized for brightness and contrast with the Gimp freeware.

Temporal variation in B frequency was assessed by contingency tables performed with the RXC program, which uses the Metropolis algorithm to obtain an unbiased estimate of the exact p-value [39]. In all cases, 20 batches of 2,500 replicates were undertaken. Spatial variation in B chromosome frequency was assessed by contingency tests comparing the six populations in 2004 and 2006 (i.e. the two years in which all the populations were sampled). To analyze prevalence, we compared B-carrying and B-lacking classes, and to compare mean B frequency we used the 0B, 1B and 2B^+^ classes. The transmission rate of B chromosomes, in controlled crosses, was analyzed by the Z-test described in López-León *et al*. [37]. Z values higher than 1.96 indicate significant drive if positive, or drag if negative.

Distance among populations and terrain topography were analyzed with the help of Google Maps. The existence of a possible inverse relationship between B_24_ frequency and distance to the center of origin (Torrox) was investigated by means of Spearman rank correlation analysis. The map in Figure 3 was obtained from OpenStreenMap.

ISSR markers were analyzed in four of the former populations (Algarrobo, Torrox, Nerja-0 and Nerja-2), and a population furthest to the east (Salobreña, Granada province), all sampled in 2006. A total of 94 ISSR fragment sizes were obtained, from six different markers, in 29 individuals from Algarrobo, 27 from Torrox, 30 from Nerja-0, 30 from Nerja-2 and 23 from Salobreña (see details in Table S1). These markers were already described in Manrique-Poyato *et al.* [14] and were analyzed as described by these authors.

Population subdivision and inbreeding analyses were performed with Hickory v1.1 [40], taking into account the dominant behaviour of these markers. The data were analyzed under four models: i) the full model, where the population subdivision (θ, analogous to Wright's *F_ST_*) and endogamy coefficient (*f*, analogous to Wright's F_IS_) were different from zero; ii) *f* = 0 model, which assumes the absence of intra-population endogamy; iii) θ = 0 model, assuming the absence of population subdivision, and iv) free model, where no previous information is assumed. The best model was chosen by the Bayesian DIC parameter (analogous to Akaike Information Criterion) [41]. This suggested that the best model was the full model, followed by the *f* = 0 model. Since the markers are dominant, however, the full model was discarded because it suggested unrealistic values for inbreeding (*f* = 0.95), which are very improbable for a polygynandric animal like *E. plorans* [42].

Population structure was analyzed by the Structure v2.3.1 software [43] using the *admixture model* with a run of 50,000 steps for burn-in and 100,000 Markov Chain Monte Carlo iterations after burnin. We determined the number of population groups (*K*) best explaining the data, following the procedure suggested by Evanno *et al.* [16], using the Structure Harvester website [44].

Isolation by distance (IBD) was investigated by means of the Mantel test between geographical distances and the *N_e_m* values obtained from pairwise *F_ST_* values provided by the AFLP-surv software [45], according to the infinite islands model [46]. The effective number of migrants per generation (*N_e_m*) was calculated by the equation: *N_e_m* = 0.25(1− *F_ST_*)/*F_ST_*. The Mantel test was performed by the Zt-win software [47] with 100,000 random permutations. Since the ISSR markers are dominant, the calculation of allele frequencies needs to assume Hardy-Weinberg equilibrium, which could lead to biased IBD estimates based on *F_ST_* values. To overcome this drawback, we used the method described by Dice [48] to calculate dis-similarity between individuals, based on the number of non-shared bands, using the FAMD v1.25 software [49]. Individual values were averaged per population to obtain a matrix of genetic distance that was compared with the geographical distance matrix by the Mantel test. Another way of testing isolation by distance was a regression of log-transformed *N_e_m* on log-transformed geographical distance values, as suggested by Slatkin [17].

To determine whether geographical barriers are reducing gene flow, we used the Barrier software version 2.2 [50], which connects the different populations by Delaunay triangulation built from the geographical coordinates of each population. The barriers are then identified using the maximum difference algorithm of Monmonier [51] to find edges associated with the highest rate of change in a genetic distance measure [50]. We used the pairwise-*F_ST_* obtained with AFLPsurv software as the genetic distance matrix. The robustness of the results was estimated from 100 bootstrapped matrices.

## Supporting Information

Figure S1 **Delta K values with respect to K, according to the calculation method posited by Evanno et al.,** [**16**] **.** These results were obtained by using all the 94 ISSR markers analyzed. Note the highest peak for K = 4.
(TIF)

Table S1 **Number of individuals analyzed for each ISSR primer.**
(DOC)

Table S2 **Proportion of individuals from each population assigned to each of the four groups (K = 4).** N =  Number of individuals analyzed. Colors refer to groups in Fig. 3a.
(DOC)

Table S3 **Pairwise matrices of geographical distance in meters (above diagonal) and** ***N_e_m*** **values (below diagonal), both transformed to log_10_.**
(DOC)
