# Supplementary figures and images for: Spread of a New Parasitic B Chromosome Variant Is Facilitated by High Gene Flow
Source: PLoS One. 2013 Dec 26;8(12):e83712. doi: 10.1371/journal.pone.0083712 (PMC3873393; doi:10.1371/journal.pone.0083712)

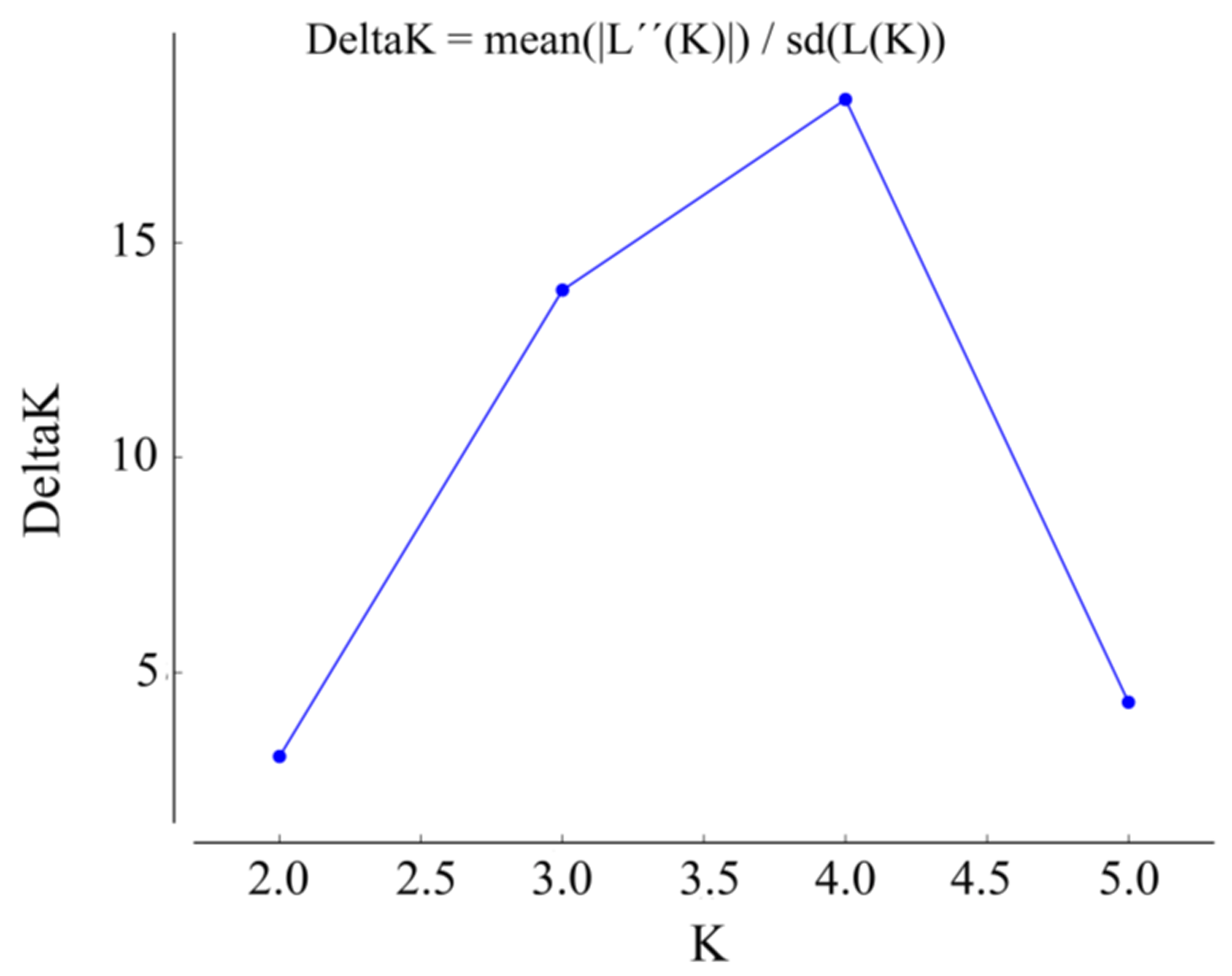

Supplement: Figure S1 — Delta K values with respect to K, according to the calculation method posited by Evanno et al., [16] . These results were obtained by using all the 94 ISSR markers analyzed. Note the highest peak for K = 4. (TIF) [file pone.0083712.s001.tif]
