# Supplementary material for: Spread of a New Parasitic B Chromosome Variant Is Facilitated by High Gene Flow
Source: PLoS One. 2013 Dec 26;8(12):e83712. doi: 10.1371/journal.pone.0083712 (PMC3873393; doi:10.1371/journal.pone.0083712)
Supplement: Table S1 — Number of individuals analyzed for each ISSR primer. (DOC) [file pone.0083712.s002.doc]

**Table S1.** Number of individuals analyzed for each ISSR primer.

|  | **Primer** | | | | | |
| --- | --- | --- | --- | --- | --- | --- |
| **Population** | **ISSR6** | **ISSR7** | **ISSR14** | **ISSR26** | **ISSR39** | **ISSR43** |
| Algarrobo | 28 | 29 | 29 | 28 | 26 | 28 |
| Torrox | 26 | 23 | 27 | 25 | 18 | 25 |
| Nerja-0 | 30 | 29 | 29 | 28 | 26 | 28 |
| Nerja-2 | 29 | 28 | 29 | 29 | 26 | 30 |
| Salobreña | 23 | 21 | 23 | 23 | 21 | 22 |
