# Supplementary material for: Spread of a New Parasitic B Chromosome Variant Is Facilitated by High Gene Flow
Source: PLoS One. 2013 Dec 26;8(12):e83712. doi: 10.1371/journal.pone.0083712 (PMC3873393; doi:10.1371/journal.pone.0083712)
Supplement: Table S2 — Proportion of individuals from each population assigned to each of the four groups (K = 4). N = Number of individuals analyzed. Colors refer to groups in Fig. 3a. (DOC) [file pone.0083712.s003.doc]

**Table S2**. Proportion of individuals from each population assigned to each of the four groups (K=4). N= Number of individuals analyzed. Colors refer to groups in Fig. 3a.

| **Population** | **N** | **Group 1 (blue)** | **Group 2 (green)** | **Group 3 (yellow)** | **Group 4(red)** |
| --- | --- | --- | --- | --- | --- |
| Algarrobo | 29 | 0.705 | 0.071 | 0.176 | 0.048 |
| Torrox | 27 | 0.290 | 0.393 | 0.294 | 0.023 |
| Nerja-0 | 30 | 0.504 | 0.034 | 0.435 | 0.027 |
| Nerja-2 | 30 | 0.348 | 0.131 | 0.495 | 0.025 |
| Salobreña | 23 | 0.029 | 0.013 | 0.016 | 0.942 |
