# Supplementary material for: Spread of a New Parasitic B Chromosome Variant Is Facilitated by High Gene Flow
Source: PLoS One. 2013 Dec 26;8(12):e83712. doi: 10.1371/journal.pone.0083712 (PMC3873393; doi:10.1371/journal.pone.0083712)
Supplement: Table S3 — Pairwise matrices of geographical distance in meters (above diagonal) and Nem values (below diagonal), both transformed to log10. (DOC) [file pone.0083712.s004.doc]

**Table S3.** Pairwise matrices of geographical distance in meters (above diagonal) and *N_e_m* values (below diagonal), both transformed to log_10_.

|  | **Algarrobo** | **Torrox** | **Nerja-0** | **Nerja-2** | **Salobreña** |
| --- | --- | --- | --- | --- | --- |
| Algarrobo |  | 3,927 | 4,119 | 4,232 | 4,608 |
| Torrox | 0,743 |  | 3,684 | 3,949 | 4,507 |
| Nerja-0 | 0,879 | 0,945 |  | 3,611 | 4,437 |
| Nerja-2 | 0,676 | 1,002 | 1,082 |  | 4,372 |
| Salobreña | 0,450 | 0,411 | 0,409 | 0,290 |  |
